# Preservative effect of Chinese cabbage (*Brassica rapa subsp*. *pekinensis*) extract on their molecular docking, antioxidant and antimicrobial properties

**DOI:** 10.1371/journal.pone.0203306

**Published:** 2018-10-03

**Authors:** Momna Rubab, Ramachandran Chellia, Kandasamy Saravanakumar, Suresh Mandava, Imran Khan, Charles Nkufi Tango, Mohammad Shakhawat Hussain, Eric Banan-Mwine Daliri, Se-Hun Kim, Sudha Rani Ramakrishnan, Myeong-Hyeon Wang, Jongkook Lee, Joong-Ho Kwon, Sangeeta Chandrashekar, Deog-Hwan Oh

**Affiliations:** 1 Department of Food Science and Biotechnology, College of Agriculture and Life Science, Kangwon National University, Chuncheon, South Korea; 2 Department of Medical Biotechnology, College of Biomedical Sciences, Kangwon National University, Chuncheon, South Korea; 3 College of Pharmacy, Kangwon National University, Chuncheon, South Korea; 4 School of Food Science and Biotechnology, Kyungpook National University, Daegu, South Korea; 5 Sri Venkateshwara Dental College, Thalambur, Chennai, Tamil Nadu, India; Tallinn University of Technology, ESTONIA

## Abstract

This study aimed at investigating the antimicrobial activity of different solvent extracts of Chinese cabbage *Brassica rapa* subsp. *pekinensis* (BRARP) and their antioxidant and cytotoxicity properties. Of the different solvents extracts, the chloroform extracts (CE) were significantly inhibited the bacterial pathogens at minimum inhibitory concentration (MIC) of 16.5 mg.mL^-1^. Biochemical analysis revealed that total phenol (62.6 ± 0.05 mg GAE.g^-1^) and flavonoids (27.6 ± 0.04 mg QE.g^-1^) were higher in the extracts of BRARP, which resulted in enhanced antioxidant activity in CE. A total of eight dominant compounds were detected in the potent antimicrobial extract from BRARP based on GC-MS analysis. The molecular interactions study revealed that, among the screened compounds the 1,2-benzenedicarboxylic acid and 2,3-dicyanopropionamide interacted with the active site of pathogenicity and survival related protein with lipopolysaccharide (LpxC) with higer binding energy. This work concluded that the 1, 2-Benzenedicarboxylic acid and 2, 3-Dicyanopropionamide from BRARP was reported to be good non-cytotoxic and antioxidant antimicrobials against bacterial pathogens.

## Introduction

*Brassica rapa* subsp. *pekinensis* (BRARP) is a vegetable crop belonging to the genus *Brassica* and the mustard family *Brassicaceae*, which is widely consumed in Asian countries, particularly in China, Korea, and Japan. The consumption of BRARP is varied widely around the world and used as a raw or steamed vegetable. Pickling (fermented food) is one of the most popular ways of preserving BRARP for preparation of dishes such as sauerkraut and kimchi. Kimchi is Korea’s representative national and traditional food and a total of about 200 types of kimchi are currently known in Korea [1]. During kimchi formation, outer leaves of BRARP are removed and inner leaves are preferred, and a greater amount of BRARP leaves thrown in the garbage. On the other hand, BRARP is rich in bioactive compounds, which are considered to have the beneficial effect for the preservation of food. Therefore, use of agro waste and their recovery into valuable products in terms of antimicrobial agents could be an economic source of antimicrobial agents for preservation of food.

The oxidative deterioration and undesirable microbial colonization in the fermented food is a major problem in food preparation during storage and distribution, such as formation of gas pockets and bloating due to excessive production of CO_2_, development of malodorous spoilage (also known as zapatera spoilage) due to decomposition of organic acids, softening of brined vegetables due to microbial pectinolytic enzymes [2]. In addition, shelf-life is also influenced by chemical changes that may result in discoloration, unpleasant taste, rancid orders, loss of quality and the formation of objectionable flavors [2, 3]. In order to avoid these problems, a wide range of chemical preservatives are used. But incorporation of preservatives can cause the several side effects in human health. Thus recent growing interest is attributed to finding the antioxidants and antimicrobial agents from the natural resource as an alternative to synthetic food preservatives. Several research reports indicated that plants and vegetables are a precious source for discovery of novel health promoting active metabolites with antioxidant and antibacterial activities [4–7]. Moreover, cruciferous vegetables are known with antimicrobial properties [8, 9].

BRARP is a natural source of nutrients and phytochemicals. Among phytochemicals, flavonoids, glucosinolate and their hydrolytic products are associated with many biological effects such as antibacterial, antifungal actions [5]. Antibacterial activity was reported to be present in BRARP and white cabbage (*Brassica oleracea var*. *capitate*) for the first time in 1936 [10] and since then it had been the subject matter of many studies in the 19^th^ century [11–14] and later on in 20^th^ century [15–16]. In the 19^th^ century itself, the destruction of antibacterial activity by heating in cabbage (*Brassica oleracea L*. *var*. *capitate*) was reported [10, 14, 17–24], but heat stable antibacterial compounds and there has been only limited information on the antibacterial activity of Chinese cabbage (BRARP).

Bacterial pathogens are a major threatening contaminant in fermented food due to its multidrug resistance, as evident by previous reports on outbreaks of *Escherichia coli* O157:H7, *Listeria monocytogens*, *Staphylococcus aureus* and *Salmonella* in the fermented food [25–27]. *E*. *coli* adapts to the acid resistance which facilitates its survival in acidic food environment of the stomach and it contributes to high pathogenicity of the outbreak strain [28]. Therefore, identification of novel antimicrobials from the natural resources against the bacterial pathogens is quite important, and their incorporation in the fermented food can increase the food shelf-life and inhibit pathogens without spoiling the nature of food. In addition, identification of potent antibacterial molecules from natural derivatives by traditional method is quite expensive and time-consuming. Hence the computer-based screening of the molecules against bacterial pathogens is quick and feasible. In this regard, we applied a computer-based molecular interactions study to reveal the inhibitory effect of metabolites from BRARP against bacteria pathogens through inhibition of Lipid A synthesizing enzyme UDP-3-*O*-(*R*-3-hydroxymyristoyl)-*N*-acetylglucosamine deacetylases (LpxC).The lipopolysaccharide (LPS) is highly charged outer membrane of Gram-negative bacteria and it is crucial for survival and pathogenicity of the bacteria which is anchor with lipid A [29–30]. The enzyme (LpxC) UDP-3-*O*-(*R*-3-hydroxymyristoyl)-*N*-acetylglucosamine deacetylase is involved in the biosynthesis of the lipid A [29–31]. The inhibition of enzyme involved in the lipid A biosynthesis can trigger entry of antibiotics towards the inhibition of bacterial cell through the down-regulation of LPS-lipid A synthesis [29, 31–32]. The targeting of the Zn+ dependent LpxC is crucial for lipid A synthesis and promising for the development of the novel antibiotics [33–34]. Hence, the present work investigated the antibacterial and antioxidant effect of various solvent extracts of BRARP, in addition to identifying the metabolites present in the solvent extracts of BRARP using GC-MS analysis. Further, interactions and binding affinity of the molecules derived from BRARP against the bacterial survival and pathogenicity related protein lipopolysaccharide (LPS) was also studied by molecular docking method. In addition, the computer-based molecular docking results were validated by *in vitro* experiments. This is the first detailed study, to our knowledge, on the antimicrobial constituents of BRARP.

## Results

### Antimicrobial activity

Antimicrobial property of different solvent extracts of BRARP was evaluated against Gram-positive bacteria, Gram-negative bacteria and fungi (Table 1). The results revealed that the chloroform, toluene, dichloromethane and ethyl ether extracts showed the antimicrobial activity while ethanol, methanol and distilled water extracts were not at all effective. Chloroform extract was the most effective among all BRARAP extracts in retarding microbial growth at the concentration of 33 mg.mL^-1^ with 13.50 to 14.50, 10.60 to 12.50 and 09.80 to 13.60 mm zone of inhibition against Gram-negative bacteria, Gram-positive bacteria, and fungi respectively. The chloroform extract had the greatest activity against Gram-negative bacteria, as well fungi except KCTC 6143 as compared to Gram-positive. While toluene extract showed the highest activity against *E*. *coli* 494 (clinical strain) and ATCC 35150 as well as ATCC 13150, and it was not at all effective with other strains tested. However, all of the extracts were not effective with no activity against KCTC 6143. DMSO and DW (used as negative control), however, data is not shown. In general, chloroform extract exhibited higher inhibitory activity than other solvents.

**Table 1:** Antimicrobial activity of different solvent extracts of BRARP.

List of microorganisms	BRARP extracts (Conc. 33 mg.mL^-1^); Zone of inhibition (mm)						
	CE	TE	DE	EEE	EtE	ME	DWE
**Gram-negative bacteria**							
494 (Isolate)	13.50 ± 0.03^a^^b^	15.00 ± 0.02^a^	13.00 ± 0.05^c^	13.10 ± 0.05^b^	-	-	-
ATCC 35150	14.50 ± 0.02^b^	18.00 ± 0.01^a^	12.50 ± 0.04^d^	13.50 ± 0.05^c^	-	-	-
ATCC 43894	14.00 ± 0.02^a^	-	12.00 ± 0.05^c^	12.50 ± 0.03^b^	-	-	-
**Gram-positive bacteria**							
ATCC 13150	12.50 ± 0.05^a^	12.00 ± 0.03^b^	10.00 ± 0.03^c^	10.00 ± 0.02^c^	-	-	-
KCTC 21004	10.60 ± 0.02^a^	-	10.60 ± 0.05^a^	-	-	-	-
KCTC 3545	11.60 ± 0.02^a^	-	-	10.60 ± 0.04^b^	-	-	-
KCTC 13302	12.30 ± 0.03^a^	-	10.00 ± 0.06^c^	10.30 ± 0.02^b^	-	-	-
**Fungi**							
KCTC 7965	13.60 ± 0.05^a^	-	11.30 ± 0.03^b^	10.30 ± 0.03^c^	-	-	-
KCTC 6145	09.80 ± 0.02^a^	-	-	08.50 ± 0.04^b^	-	-	-
KCTC 6143	-	-	-	-	-	-	-
KCTC 6317	13.60± 0.02^a^	-	10.60± 0.05^b^	-	-	-	-

-: not active, CE: Chloroform Extract, TE: Toluene Extract, DE: Dichloromethane Extract, EEE: Ethyl Ether Extract, EtE: Ethanol Extract, ME: Methanol Extract, DWE: Distilled Water Extract

^a^: more sensitive

^b^: moderate sensitive

^c^: less sensitive, MHA-Muller Hinton Agar, MRS-De Man, Rogosa and Sharpe agar

The antimicrobial activity of BRARP was compared with the standard antibiotics as shown in S1 Table. Ampicillin, kanamycin and tetracycline were highly effective against the tested bacterial strains, while penicillin and gentamicin were found have inhibitory activity, similar to BRARP extract (S1 Table). Vancomycin and erythromycin were least sensitive towards the bacterial growth, however, clindamycin and Novobiocin displayed variable trend. In general, all the tested antibiotics showed similar zone of inhibition except ampicillin, tetracycline, and kanamycin. In terms of comparison with chemical preservatives, only sodium metabisulphite was effective against all the microorganisms tested in this study at the permissible limit in food, as shown in S2 Table. Thus BRARP extract could be an attractive alternative for the preservation of food as a natural antimicrobial agent.

#### Thermostability

Thermostability of the antimicrobials extracted in chloroform, dichloromethane, and ethyl ether was determined by disc diffusion method after heating at 95°C for six different time intervals (5, 15, 30, 45, 60 and 90 min). All the heat treatments showed the inhibitory activity against the microorganisms tested, however, the activity was reduced after heat treatment, as shown in Table 2. All the heat treated samples showed activity, and hence the compounds responsible for the antimicrobial activity might not be protein in nature. It also revealed the stability of compounds at higher temperature and showed the activity even after 90 min of heating at 95°C. We also found even after enzyme treatment, the samples exhibited antimicrobial activity, as shown in S3 Table.

**Table 2:** Thermostability of chloroform extract of BRARP.

List of microorganisms	Chloroform extract at 95°C at different time periods (Min); Zone of inhibition (mm)					
	5	15	30	45	60	90
**Gram-negative bacteria**						
494 (Isolate)	11.00 ± 0.01^b^	11.00 ± 0.03^b^	10.50 ± 0.05^b^^c^	12.00 ± 0.01^a^	11.00 ± 0.04^b^	11.00 ± 0.03^b^
ATCC 35150	13.00 ± 0.02^a^	12.00 ± 0.01^b^	11.00 ± 0.03^c^	13.00 ± 0.02^a^	10.00 ± 0.03^d^	12.00 ± 0.01^b^
ATCC 43894	12.00 ± 0.02^b^	14.00 ± 0.03^a^	10.00 ± 0.03^c^	12.00 ± 0.02^b^	12.00 ± 0.05^b^	14.00 ± 0.03^a^
**Gram-positive bacteria**						
ATCC 13150	10.50 ± 0.01^a^	09.50 ± 0.03^b^	08.00 ± 0.02^d^	10.50 ± 0.01^a^	09.00 ± 0.05^c^	09.50 ± 0.03^b^
KCTC 21004	10.00 ± 0.02^a^	09.00 ± 0.03^b^	09.00 ± 0.01^b^	10.00 ± 0.02^a^	09.00 ± 0.03^b^	09.00 ± 0.03^b^
KCTC 3545	10.00 ± 0.02^a^^b^	10.30 ± 0.05^a^	10.00 ± 0.02^a^^b^	10.00 ± 0.02^a^^b^	10.00 ± 0.03^a^^b^	10.30 ± 0.05^a^
KCTC 13302	10.00± 0.02^a^	09.00± 0.03^b^	10.00± 0.03^a^	10.00 ± 0.02^a^	10.00± 0.05^a^	09.00 ± 0.03^b^
**Fungi**						
KCTC 7965	10.00 ± 0.03^a^	09.00 ± 0.01^b^	10.00 ± 0.03^a^	10.00 ± 0.03^a^	10.00 ± 0.04^a^	09.00 ± 0.01^b^
KCTC 6145	09.00 ± 0.02	08.00 ± 0.03	08.30 ± 0.03	09.00 ± 0.02	09.00 ± 0.03	08.00 ± 0.03
KCTC 6143	-	-	-	-	-	-
KCTC 6317	12.30 ± 0.02^a^	12.30 ± 0.03^a^	12.00 ± 0.01^a^^b^	12.30 ± 0.02^a^	12.00 ± 0.04^a^^b^	12.30 ± 0.03^a^

-: not active

^a^: more sensitive

^b^: moderate sensitive

^c^: less sensitive, MHA-Muller Hinton Agar, MRS-De Man, Rogosa and Sharpe agar

#### Minimum inhibitory and bacteriostatic concentration

**Fig 1:**
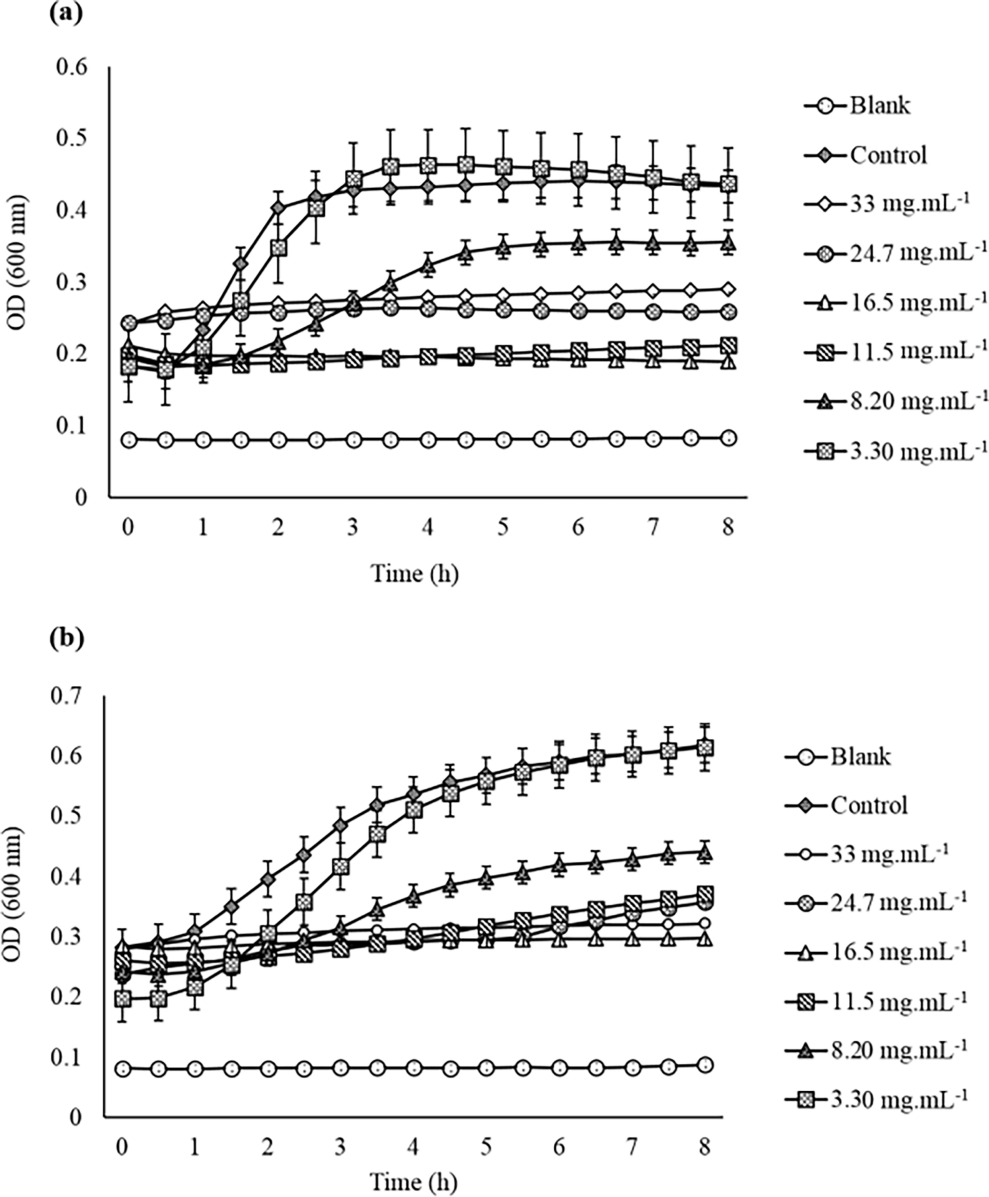
Determination of minimum inhibitory concentration based on growth curve assay of BRARP crude extract, 1a: ATCC 35150, and 1b: ATCC 13150, TSB: Tryptic Soy Broth.

The MIC of the most effective BRARP chloroform extract was employed by disc diffusion method and the concentration-dependent effect of the extract is shown in Fig 1A and 1B. The inhibitory effect of BRARP extracts was started at 11.5 mg.mL^-1^ with an inhibition zone of 8.5 and 9.0 mm against ATCC 13150 and ATCC 35150, respectively. The MBC was confirmed by growth inhibitory activity against ATCC 35150 and ATCC 13150 for 8 h using spectrophotometer at 600 nm and 37°C. The results indicated that BRARP potentially bacteriostatic against the pathogenic bacteria (Fig 1A and 1B). The MBC of the extract was 16.5 mg.mL^-1^ for ATCC 35150 and ATCC 13150.

### Antioxidant activity

**Fig 2:**
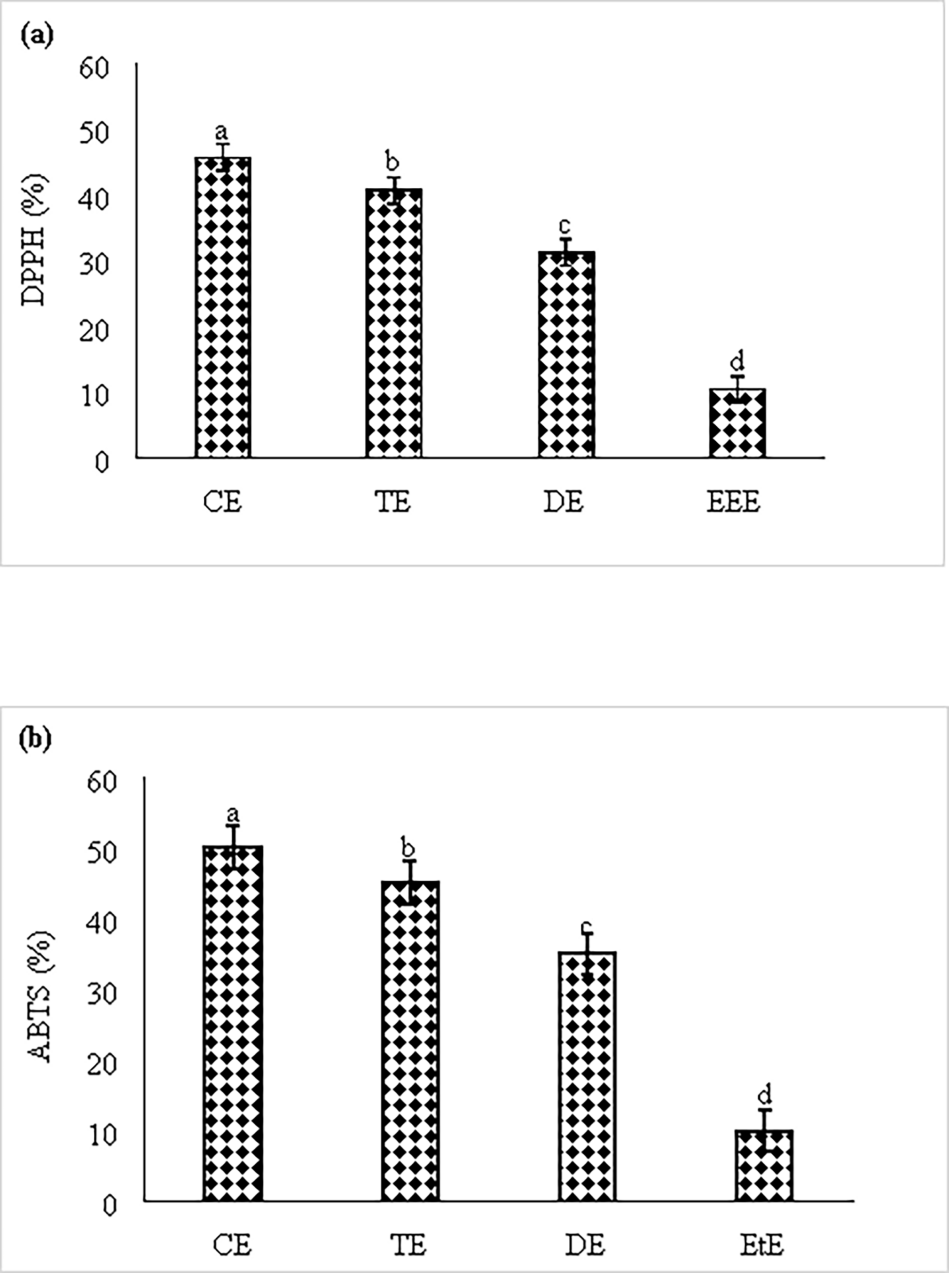
Antioxidant activity of different extracts of BRARP, (a) DPPH and (b) ABTS assay.

Antioxidant property of different solvent extracts of BRARP was investigated by measuring 2, 2-diphenyl-1-picrylhydrazyl (DPPH) free radical scavenging activity and ABTS assays, and the results are shown in Fig 2A and 2B. The sample was tested at different concentrations ranging from 50 to 800 μg.mL^-1^ (Fig 2A and 2B). The 800 μg.mL^-1^ of chloroform extract (CE) of BRARP showed the highest DPPH radical scavenging activity (45.75%) (Fig 2A). As compared with chloroform extract, low activity was observed for all other solvent extracts (31.25% DE, 10.50% EtE and 40.75% TE). ABTS assay also showed the similar trend of antioxidant activity in CE, DE, TE and EtE with 50, 45, 35 and 10%, respectively. While, no scavenging activity was observed in methanol, ethyl ether, and distilled water extracts in both antioxidant assays.

### Phytochemical analysis

**Fig 3:**
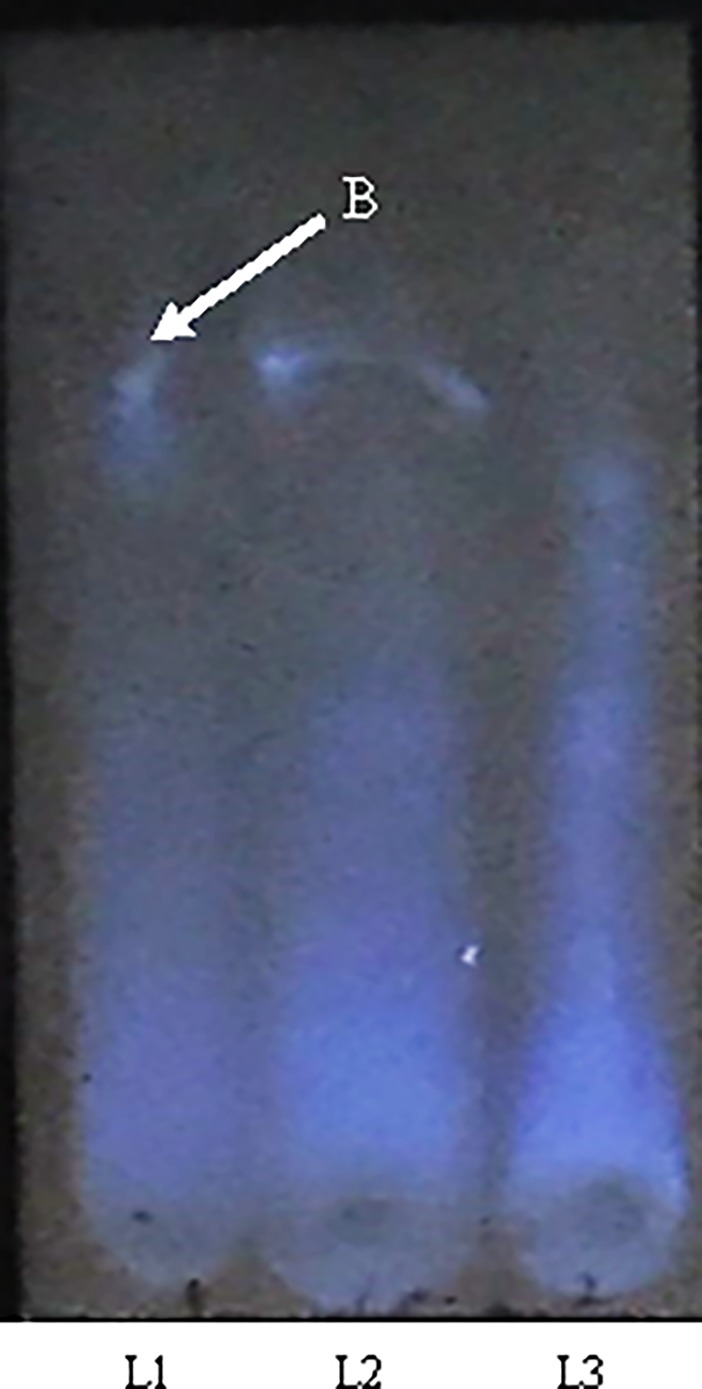
HPTLC chromatogram of phenolic acid fractions from BRARP migration distance: 40 mm; application volume, polyethylene glycol reagent (NP/PEG) (Fluka Chemie, Switzerland). UV254 nm light detection; (c) UV365 nm light detection. Bands (B): 1, Phenol. Lanes (L): 2. Chloroform extract BRARP of (33 mg.mL^-1^); 3. Chloroform extract of BRARP (16.5 mg.mL^-1^).

The presence of biochemical compounds in the different solvent extracts of BRARP was analyzed. The results indicated the presence of saponin in chloroform and dichloromethane extracts, and steroids in ethyl ether and toluene extracts. While glycosides was present in all the extracts (except ethanol and methanol extracts) and none of compounds was found in methanol and ethanol extracts (S4 Table). Quantitatively 62.6 ± 0.05 mg GAE.g^-1^ of total phenol and 27.6 mg QE.g^-1^ of total flavonoid were recorded in chloroform extract of BRARP. While using two chromatography solvents as mobile phase, ethyl acetate/formic acid/acetic acid/water, 100:11:11:26 (*V/V*) showed good separation of phenols, as shown in Fig 3. Among the extracts of BRARP, chloroform extract exhibited the presence of phenol by blue band.

### GC-MS analysis

The extract was subjected to phytochemical analysis using optimized GC-MS parameters and the resultant gas chromatograms are presented in S2 Fig and S3 Fig for chloroform and methanol extracts of BRARRP, respectively. In total, eight bioactive compounds were identified from the crude extract of BRARP in both the solvents and relative percentage of peaks of the compounds. Putative empirical formulas and identifications were obtained for all of the identified compounds by comparison with the database.

The Antimicrobial activity of the identified commercially available compound was tested using disc diffusion method as shown in S5 Table. (E)-2-Butenoic acid propyl ester, phenol, sodium phenoxide, and 4-Pyridinecarboxylic acid were not at all effective against the microorganisms tested even at higher concentration of 1 mg.L^-1^. The 2, 2-dimethoxybutane and s-Triazolo [4, 3-a] pyridazine were effective against three microorganisms, but not against the fungal strains. But 1,2-Benzenedicarboxylic acid showed comparatively efficient antagonist activity against all the microorganisms tested. At low concentration (0.5 mg.mL^-1^), this compound was less sensitive as compared to BRARP extract however, at high concentration (1 mg.mL^-1^) it showed better activity with comparable zone of inhibition (S5 Table).

### Cytotoxicity

The cytotoxic effect of the commercially available identified compounds against MCF-7 cell line was determined using the MTT assay to compare with BRARP extracts. The results are summarized in S6 Table. None of the commercially available compounds exhibited considerable cytotoxicity against MCF-cell line after 48 h of incubation Table 3. However, 1, 2 Benzenedicarboxylic acid and 2, 3- Dicyanopropionamide exhibited slight cytotoxic effect after 48 h of incubation with 44.3 and 41 μg.mL^-1^, respectively.

**Table 3:** Cytotoxic activity of BRARP in different solventsagainst MCF -7 cell line.

Sr. No	Plant Extract	IC_50_ (μg.mL^-1^)
		MCF-7
1	*Brassica rapa* subsp. *pekinensis* (BRARP)*	>50
2	*Brassica rapa* subsp. *pekinensis* (BRARP)**	>50
3	*Brassica rapa* subsp. *pekinensis* (BRARP)***	>50
4	*Brassica rapa* subsp. *pekinensis* (BRARP)****	>50
5	*Brassica rapa* subsp. *pekinensis* (BRARP)*****	>50
6	*Brassica rapa* subsp. *pekinensis* (BRARP)******	>50
7	Tamoxifen	10.58

-: *EEE: Ethyl ether Extract

**TE: Toluene Extract

***EtE: Ethanol Extract

****ME: Methanol Extract

*****CE: Chloroform Extract

******DE: Dichloromethane Extract, IC: Half maximal inhibitory concentration, MCF: Michigan Cancer Foundation-7 (Brest cancer cell line)

### Molecular interaction

**Fig 4:**
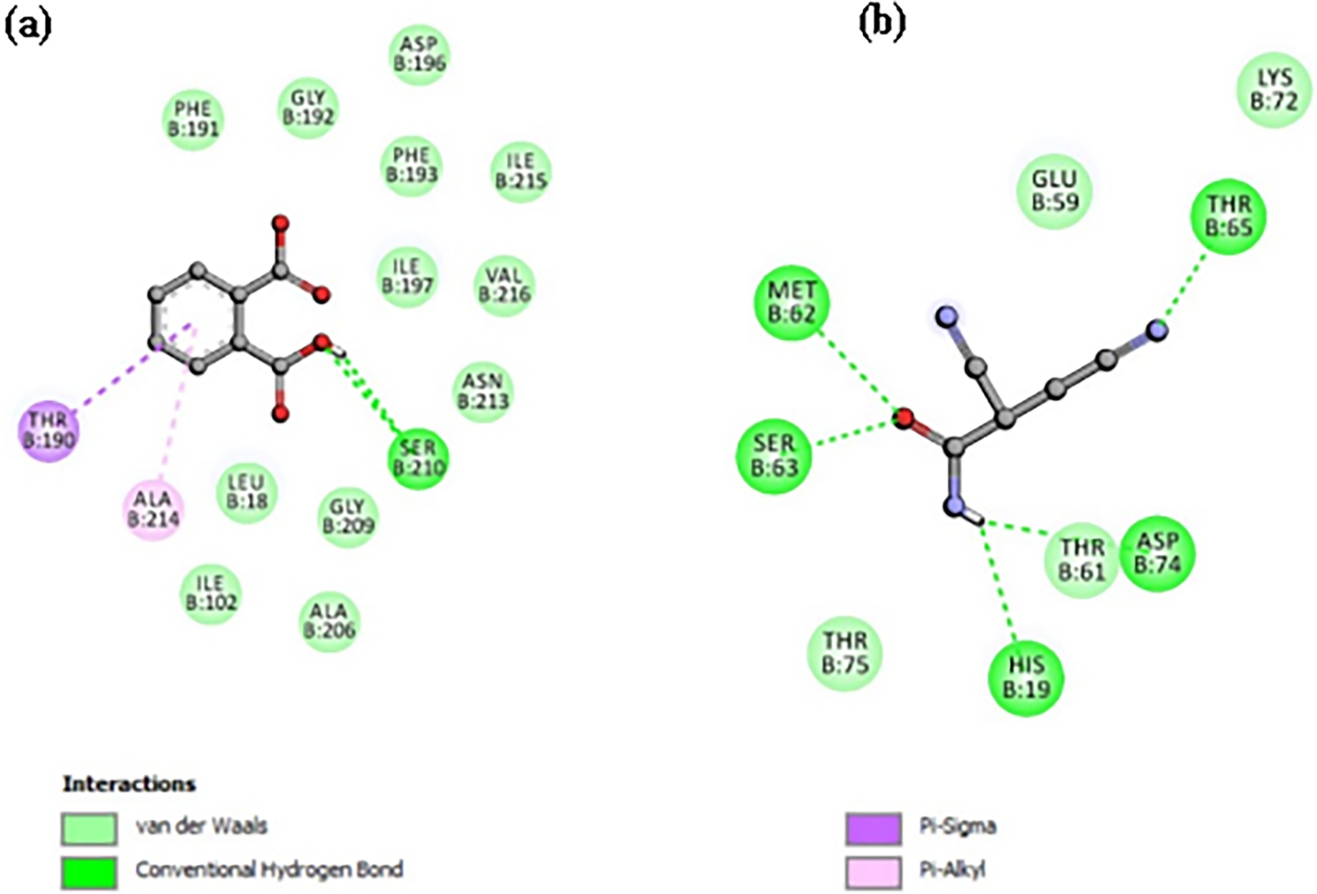
Predicted binding mode complex of molecular model (a) 1,2-Benzenedicarboxylic acid and LpxC complex, (b) 2,3-Dicyanopropionamide and LpxC complex.

**Fig 5:**
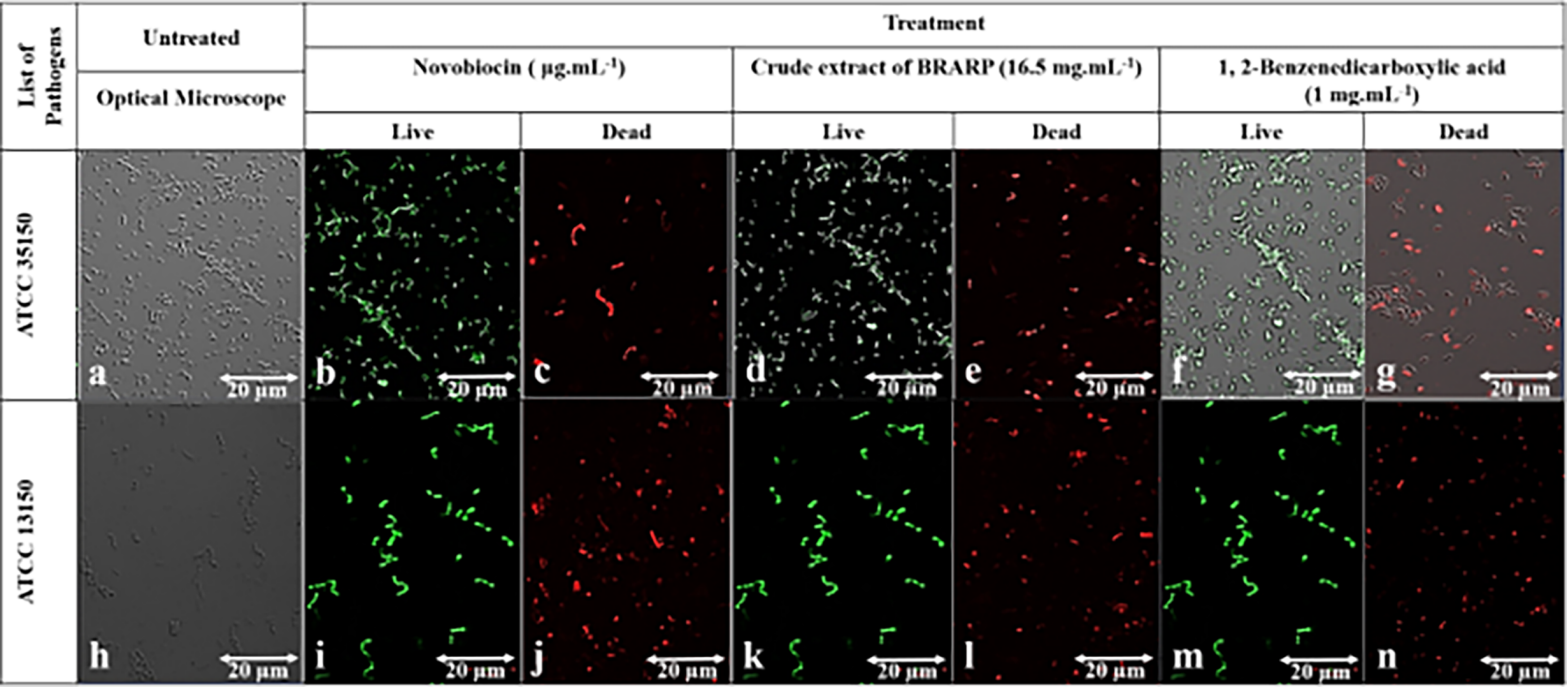
Adhered of dead/live cells Confocal Microscopic imaging of ATCC 35150 and ATCC 13150 in which observed green spots (Syto-9) healthy live cells and red spots (Propidium Iodide) dead cells (20X magnification). (a, h) optical microscopic observation; (b, d, f) untreated ATCC 35150; (c) ATCC 35150 treated with Novobiocin; (e) ATCC 35150 treated with BRARP extract; (g) ATCC 35150 treated with 1, 2-Benzenedicarboxylic acid 1.0 mg.mL^-1^; (I, k, m) untreated of ATCC 13150; (j) ATCC 13150 treated with Novobiocin, (l) ATCC 13150 treated with BRARP extract; (n) ATCC 13150 treated with 1, 2-Benzenedicarboxylic acid 1.0 mg.mL^-1^.

Among all identified compounds, 1,2-Benzenedicarboxylic acid and 2,3-Dicyanopropionamide inhbitied the LpxC as evident by their higher ability with better docking score of -5.8 and -4.8 Kcal.mol^-1^ to fit the catalytic active site of LpxC than the other molecules tested.The results are shown in Table 4 and Fig 4A and 4B, and other weaker inhibiting molecules and their interactions shown in S1 Fig. 1,2-Benzenedicarboxylic acid was fit the active catalytic site of LpxC and established the strong interactions with hydrogen bonds of Ser 210 also with pi Alkyl and sigma residues of Ala 214 and Thr190 as well as van der waals of Phe191, Gly 192, Asp 196, Phe 193, Ile197, 215, Val 216, Asn 213, Gly 209, Leu 18, Ile 102. Whereas the 2,3-Dicyanopropionamide established the strong interactions with the catalytic site of LpxC via hydrogen bonds residues such as Thr 65, Asp 74, His 19, Ser 63, Met 62 as well van der vaalsThr75, Glu 59, Thr 61, and Lys 72. This molecular interaction study revealed that 1, 2-Benzenedicarboxylic acid was potentially involved in inhibition of bacterial pathogens through inactivation of LpxC. These results were further validated by *in vitro* antibacterial assay using the pure compound of 1,2-Benzenedicarboxylic acid and adhered live and dead bacterial cells by confocal imagining before and after treatment (Fig 5). There was no significant difference in the number of live and dead cells between the extract and 1,2-Benzenedicarboxylic acid (Phthalic acid) treated one.

**Table 4:** Molecular docking score of identified metabolites from chloroform and methanol extract of BRARP.

Name of the compound	Chemical formula	Molecular weight (Da)	Area (%)	Docking score (Kcal.mol^-1^)	Activity	References
**Chloroform extract**					** **	
(E)-2-Butenoic acid propyl ester	C_7_H_12_O_2_	128.169	0.05	-4	ACE, Angiotensin-converting enzyme	[36, 37]
Phenol	C_6_H_6_O	94.111	0.05	-4.5	Antimicrobial	[37–38]
Sodium phenoxide	C_6_H_5_NaO	116.093	0.06	-4.1		
4-Pyridinecarboxylic acid	C_6_H_5_NO_2_	123.109	0.05	-4.6	Anticancer (oral), Antidote, Orexigen	[39]
s-Triazolo[4,3-a]pyridine	C_6_H_5_N_3_	119.124	0.02	-4.6	Antimicrobial	[40]
1,2-Benzenedicarboxylic acid	C_8_H_6_O_4_	166.131	0.01	-5.8	Antimicrobial, antioxidant	[41, 42]
**Methanol extract**					** **	
2,2-Dimethoxybutane	C_6_H_14_O_2_	118.174	0.04	-3.5	Antimicrobial,	[43, 44]
2,3-Dicyanopropionamide	C_5_H_5_N_3_O	123.113	1.19	-4.8	Antimicrobial	[45]

## Discussion

Antimicrobial properties of plant materials are being increasingly reported from different parts of the world. The plant extract derived active constituents are used in traditional therapies for 80% of the world’s population [4]. The emerging antimicrobial resistance in pathogens, mainly due to continuous practice of antibiotics, it can be resolved based on antimicrobial compounds present in secondary metabolites extracted from the natural plant materials [5, 6]. Much attention is being paid towards plant extracts and biologically active compounds isolated from natural resources [4, 35]. Plants are rich source of structurally novel and biologically active metabolites. Secondary metabolites present in the plants may be potential bioactive compounds of interest in the food industry for preservation of food. Most of the secondary metabolites present in plants have antimicrobial activity, but their efficiency differs based on the compound and the target pathogens [16]. In the present work, the extracts obtained from BRARP showed strong antimicrobial activity against MDR bacteria (Isolate (494), ATCC 43894, ATCC 35150, and ATCC 13150) and fungi (KCTC 7965, KCTC 6143, KCTC 6145 and KCTC 6317) which are most challenging organisms in the safety of products.

In the present study, mid-polar extracts exhibited maximum antimicrobial activity, compared to other solvent extracts by disc diffusion method. Although this method is sensitive to detect microbial growth, it is only a qualitative test and should not be endorsed to quantify the antimicrobial activity of a substance based on the zone of inhibition formed [36]. Anyhow, all the BRARP extracts were tested in disc diffusion method indicated the potential of antimicrobial activity, however, chloroform extract of BRARP had the most effective activity. Several previous reports are in support of the present findings of antimicrobial activity of different kinds of cabbages [16, 37–38]. Among the pathogenic microbes tested, only one fungus strain (KCTC 6143) was not susceptible to BRARP extract. The results showed BRARP extract was more effective against the Gram-negative bacteria (Isolate (494), ATCC 43894, and ATCC 35150) than Gram-positive (ATCC 13150) and LAB strains (Table 1). Similar findings were observed with the extracts of different berries such as blueberry, raspberry, and strawberry [31, 39]. BRARP exhibited the inhibitory activity against the lactic acid bacteria, but it was not well pronounced and just suppressed the activity and hence it could be helpful to preserve the fermented food to control fermentation process after opening of the product. In this screening work, extracts of BRARP were found to be not inactive against any organisms, such as Gram-positive, Gram-negative, Lactic acid bacteria and fungi strains. In comparison with commercially available antibiotics, some of the antibiotics such as clindamycin and erythromycin were not effective at all against the microorganisms tested in this study, however, other antibiotics showed the strong inhibitory activity as compared to BRARP (S1 Table). Efficacy of plants can be affected by many factors. For instance, different plants have differences in combinations of secondary metabolites such as phenolic compounds, tannins, alkaloids and steroids [40]. These metabolites are also deposited in varying proportions in different parts of an individual plant. Differences in solvents used for extraction as well as geographical location could also have contributed to observed variations. This could explain lower activity of the BRARP extracts compared to the commercially available antibiotics tested in this study. These results suggest that BRARP is a potential source of broad-spectrum antimicrobial agents. In the present study, the BRARP chloroform extract was subjected to heat treatment at 95°C for different time intervals (5, 15, 30, 45, 60 and 90 min), which resulted in a decrease in antimicrobial activity, however, difference was not significant. This is in agreement with the previously report [41].

As shown in Table 1, chloroform extract of BRARP was the most effective in inhibiting Gram-positive bacteria and Gram-negative bacteria with minimum inhibitory concentration (MIC) value of 11.5 to 16.5 mg.L^-1^. The MIC is defined as the lowest concentration that completely inhibits growth of the microorganisms.

In this study, the commonly accepted assays viz 1,1-diphenyl-2-picrylhrdrazyl (DPPH) and ABTS radical cation decolorization assay were used to evaluated the antioxidant activity of BRARP with different solvents. The total antioxidant, measured by DPPH and ABTS method, ranged from 10.50 to 45.75% and 10 to 50% respectively. The results from the antioxidant assay showed that BRARP extracts could scavenge the radical to a certain extent. This is accordance with previous report [34]. The reducing power of BRARP extracts varied markedly with different solvents (Fig 2A and 2B). This work demonstrated under DPPH and ABTS assays, that some of extracts had reducing power and the power in descending order among those extracts was chloroform > toluene > dichloromethane > ethanol. The values for all extracts of BRRAP with different solvents obtained by ABTS assay were higher than those obtained by DPPH assay. The phenolic compounds present in BRARP chloroform extracts showed a strong positive correlation with the free radical scavenging activity.

This study revealed the presence of polyphenols, saponin, glycosides, steroids, and flavonoids in BRARP extract, similar to previous studies [37–42]. Further, thin layer chromatography (TLC) revealed the presence of phenols in chloroform extract of BRARP, akin to earlier work [43]. Among seven solvents (chloroform, dichloromethane, toluene, ethanol, methanol and distilled water), chloroform was found most effective in extracting secondary metabolites.

The GC-MS analysis of the BRARP extract detected a number of interesting compounds. Some of these compounds have earlier been reported to possess anticancer, antioxidant and antimicrobial potentials (Table 4) [44–53]. The present study further found the antimicrobial activity of commercially available identified compounds and compared. 1, 2-Benzenedicarboxylic acid showed antimicrobial activity in terms of zone of inhibition, which was compared with BRARP extract. This compound is earlier reported to have antimicrobial activity [50–54].

The cytotoxicity effect was evaluated by conducting MTT cell viability assay, using tamoxifen as the standard. Table 4 revealed that all extracts of BRARP showed no significant cytotoxicity with IC_50_ value of only >50 μg.L^-1^ against MCF-7 cell line. This finding is in agreement with the previous report on *Brassica rapa* ssp. *Campestris* [55].

Further, to clarify the antimicrobial potential of the identified compounds in BRARP, the molecular docking analysis was performed in order to validate the obtained data and to provide understandable evidence for the observed antimicrobial activity of all identified compounds in GC-MS analysis. The molecular docking is a well-established technique to determine the interaction of two molecules and to find out the best orientation of ligand to form a complex with overall minimum energy. The result of molecular docking was successfully validated by laboratory assay (Table 4). The ligand binding landscape and its interaction pattern were analyzed from the top ranked binding pose. Survival of bacteria is strongly linked with the Lipid A biosynthetic pathway, which is functioning through the UDP-3-*O*-(*R*-3-hydroxymyristoyl)-*N*-acetylglucosamine deacetylases (LpxC). To identify the antibiotic molecule against bacterial infection, the LpxC was targeted in the molecular docking. The docking analysis revealed that all the identified compounds showed good binding energy towards the target LpxC site ranging from -3.5 to -5.8 Kcal.mol^-1^. According to GC-MS analysis 2, 3-Dicyanopropionamide showed the highest peak area with 1.19% as compared to 1,2-Benzenedicarboxylic acid with 0.01%. However, molecular docking analysis score showed strong binding efficiency for 1,2-Benzenedicarboxylic acid as compared to 2, 3-Dicyanopropionamide docking score. Hence this study suggested for purification or biological synthesis of 1,2-Benzenedicarboxylic acid and 2,3-Dicyanopropionamide towards the development of molecular leads for antibacterial drugs against bacterial infections.

The present study provides the useful information about antimicrobial and antioxidant properties and polyphenolic contents of BRARP, which is commonly used for fermented food purpose. This work demonstrated that mid-polar extracts of BRARP were a potential source of polyphenols with significant antimicrobial activity. The MIC was determined to be 16.5 mg.mL^-1^ against tested microorganisms and the compounds responsible for the antimicrobial activity of BRARP were 1, 2-Benzenedicarboxylic acid and 2,3-Dicyanopropionamide, as confirmed by molecular modeling followed by *in vitro* laboratory experiments. The identified compounds can be utilized for the development of natural antimicrobial agent against pathogenic bacteria for the preservation of food. Therefore, the docking studies have widened the scope of developing a new class of antimicrobial agents. Moreover, BRARP extract could be considered as an eco-friendly and cost-efficient alternative as compared to the synthetic preservatives for food.

## Materials and methods

### Materials

Ampicillin, gentamicin, tetracycline, erythromycin, Novobiocin, pancreatin, Folin-Ciocalteu’s phenol reagent, (2 N), 1, 1 diphenyl-2-picrylhrdrazyl, sodium metabisulphite, (E)-2-Butenoic acid propylester, phenol, sodium phenoxide, 4-Pyridinecarboxylic acid, 1,2-Benzenedicarboxylic acid (Phthalic acid) and 2,3-Dicyanopropionamide were purchased from Sigma-Aldrich, South Korea, kanamycin from Carl ROTH, South Korea. 2, 2-dimethoxybutane and s-Triazolo[4, 3-a] pyridine were purchased from Matrix Science, United States. Vancomycin, penicillin, and clindamycin were procured from Gold Bio Technology, Inc. South Korea. Natural products-polyethylene glycol reagent (NP/PEG) was purchased from Duksan Science, South Korea. MRS agar and MRS broth were purchased from MB cell, South Korea. Nutrient broth was purchased from Becton, Dickinson and Company, United States, and Muller-Hinton agar from Thermo Fisher Scientific, Gangnum-gu Seoul, Korea. Chloroform dichloromethane, toluene, sulphuric acid, ascorbic acid, quercetin were purchased from Junsei Chemical Co., Ltd. South Korea. Anhydrous ethyl alcohol, methyl alcohol, ethyl ether, toluene and ethyl acetate were purchased from Daejung chemicals & metals Co., Ltd, Gyeonggi-do, South Korea. Ferric chloride, acetic acid, gallic acid, aluminum chloride, sodium bicarbonate, sodium nitrate, sodium nitrite, Dimethyl sulfoxide (99.5%) were purchased from Biosesang Gyeonggi-do, South Korea. Formic acid (98.05) from Kanto Chemical Co., Inc. South Korea.

Microbial strains used in this study were Gram-positive bacterial pathogens (*Staphylococcus aureus* ATCC 13150, *Lactobacillus plantarum* KCTC 21004, *Lactobacillus helveticus* KCTC 3545 and *Leuconostoc mesenteroides* KCTC 13302), three Gram-negative bacterial pathogens (*Escherichia coli* 494 (isolate), ATCC 35150 and ATCC 43894) and four fungal pathogens (*Candida albicans* KCTC 7965, *Aspergillus fumigatus* KCTC 6145, *Aspergillus flavus var*. *flavus* KCTC 6143 and *Aspergillus niger* KCTC 6317). The fungal and lactic acid bacterial (LAB) strains were obtained from Korean Collection for Type Cultures (KCTC). *E*. *coli* ATCC 43894 and ATCC 35150 strain and *S*. *aureus* (ATCC 13150) were obtained from American Type Culture Collection (ATCC) and *E*. *coli* 494 was received from U.S Food Fermentation Laboratory Culture Collection (USDA ARS, Raleigh, N.C., U.S.A) and USDA ARS Eastern Regional Research Center (Wyndmoor, Pa., U.S.A).

#### Preparation of Chinese cabbage (BRARP) Extracts

Fresh *B*. *rapa subsp*. *Pekinensis* was (BRARP) purchased from a local supermarket in Chuncheon, South Korea and washed with distilled water. Then the leaves and stem were chopped with a knife and dried in an oven at 75°C for 2 days. The dried samples were crushed into a fine powder using an electrical blender and stored at room temperature until used for extraction. For extraction, 2 g of the dried powder was added into to each glass bottle containing 50 mL of ethyl ether, toluene, ethanol, methanol, chloroform, dichloromethane and distilled water separately and subjected to extraction by shaking at 37°C with 200 rpm for 24 h. Then the solvents were evaporated in open air for 48 h. After evaporation, the samples were eluted by using water for polar and dimethyl sulfoxide for non-polar extracts by agitation for 3 h on a magnetic stirrer. Afterwards, the solutions were centrifuged at 3000 rpm for 20 min and the supernatant was separated from the solid material and kept it in a refrigerator for further antimicrobial assay.

### Antimicrobial activity

#### Inoculum preparation

Each bacterial strain was grown in selective broth (nutrient broth for ATCC 13150 and isolate (494), ATCC 43894, ATCC 35150 strains) at 37°C with 150 rpm for 16–18 h. The LAB and fungal strains were grown in MRS broth at 30°C with 150 rpm for 16–18 h. The bacterial and fungal cells were harvested using 0.1% sterilized buffered peptone water, its absorbance was adjusted to 600 nm and diluted to attain viable cell count of 10^8^ CFU.mL^-1^ using a spectrophotometer.

#### Antimicrobial assay

The disc diffusion method [56–57] was adapted to evaluate the antimicrobial activity of BRARP extracts. Each microbial suspension (100 μL) was inoculated onto Muller-Hinton and MRS agar surface using a spreader. Sterile filter paper discs of 8 mm in diameter each, loaded with BRARP extracts (33 mg.mL^-1^) were aseptically placed on the top of agar surface. The inoculated plates were allowed to stand at ambient temperature for 30 to 45 min to allow diffusion of extracts prior to incubation at 37°C for Gram positive bacteria and Gram negative bacteria and 30°C for lactic acid bacteria and fungi for 24 h. Control experiments were carried out under same conditions by using DMSO and DW as negative control and antibiotics (ampicillin, vancomycin, penicillin, gentamicin, tetracycline, kanamycin, clindamycin, erythromycin and Novobiocin) and chemical preservatives (sodium metabisulphite, sodium nitrate, and sodium nitrite) as positive control. The zone of inhibition was observed and measured after 6 h of incubation. A positive result was defined as an inhibition zone of ≥ 9 mm indicating the presence of antimicrobial substances in the extracts tested [58].

#### Thermostability

The thermostability of the BRARP chloroform extract was determined by disc diffusion method. The extract was heated at 95°C for six different time intervals (5, 15, 30, 45, 60 and 90 min) and stored at 4°C until use. Then antimicrobial activity was evaluated by disc diffusion method as described in section 2.3.3 [41].

#### Pancreatic enzyme treatment

Pancreatic enzyme treatment was performed to determine the protein nature of the bioactive compound. The assay was performed by treating the plant BRARP extract with a pancreatic enzyme (1 mg.mL^-1^), incubated for 2 h at 37°C and then heated at 85°C for 20 min to denature (deactivate) the enzyme. Then antimicrobial assay was performed by disc diffusion method as described in 2.3.3 against all test microorganisms used in this study.

#### Minimum inhibitory concentration (MIC)

MIC is defined as the lowest concentration of antimicrobial agent that inhibits the microbial growth after 24 h of incubation. The most effective BRARP extract which exhibited the strong antimicrobial activity at 16.5 mg.mL^-1^ was manipulated to determine their MIC using disk diffusion method and evaluated their efficiency towards entero-pathogens. Different concentrations of BRARP extract (3.30, 8.20, 11.5, 16.5, 24.7 and 33 mg.mL^-1^) were prepared separately in methanol and performed the antimicrobial activity by disc diffusion method as described in 2.3.3.

#### Growth inhibitory activity

The growth inhibitory activity of the BRARP extract was determined against ATCC 35150 and ATCC 13150 by using 96-well microtiter plate. The bacterial concentration with 10^2^ CFU.mL^-1^ was mixed with different concentrations of the extract and make the final volume up to 200 μL and inhibitory activity was measured at 600 nm for 8 h at 37°C by using a spectrophotometer. Bacteriostatic or bactericidal effects were determined, based on growth inhibition. Further the morphological changes, dead and live cells of bacterial cells were examined under super sensitive high resolution confocal laser scanning microscope imaging (SR-CLSM; LSM880 with Airyscan, ZEISS, Oberkochen, Germany) after staining the live/dead cells with Syto-9 (Laser Line- 488nm; Excitation -617; Emission- 503) and propidium Iodide (Laser Line-488nm; Excitation-535; Emission-488) respectively.

#### Antioxidant activity assay

Antioxidant property of BRARP extract was investigated by DPPH and ABTS free radical assay following the previously reported methods with slight modifications [59, 60].

#### Analysis of Secondary metabolites/volatile compounds

The extracts were subjected to preliminary secondary metabolites testing to detect for the presence of different chemical groups of compounds. The oven-dried crude extract was screened for the presence of phenol, saponins, tannins, flavonoids, terpenoids, steroids, glycosides as according to the methods described elsewhere [61, 62].

#### Qualitative analysis of Flavonoids

Thin-layer chromatography (TLC) was performed on pre-coated 20 x 20 cm TLC plates coated with 0.25 mm layers of silica gel 60 F254 (Merck). After application of the extract and standard solutions (10 L), the plates were developed for 19 cm in paper-lined all-glass chambers (Desaga, Germany) previously left to equilibrate for at least 30 min. Two chromatography solvents were used: ethyl acetate/formic acid/acetic acid/water, 100:11:11:26 (*V/V*) and ethyl acetate/formic acid/water, 8:1:1 (*V/V*) [35, 43]. Visualization of the flavonoids and phenolic acids was achieved by spraying the sheets with natural products-polyethylene glycol reagent (NP/PEG) (Fluka Chemie, Switzerland). Typical intense fluorescence in UV light at 365 nm was produced immediately on spraying (flavonoids appeared as orange-yellow bands, whereas phenolic acids formed blue fluorescent zones). Addition of polyethylene glycol solution lowered the detection limit and intensified fluorescence.

#### Total flavonoid content (TFC)

TFC was determined by a spectrophotometric method with slight modifications [63]. Briefly, 250 μL of the extract was mixed with 100 μL of 2% AlCl_3_ solution (prepared in methanol) and incubated for an hour at room temperature and the absorbance was measured by using spectrophotometer at 415 nm. The same procedure was repeated for the standard solution of quercetin of different concentrations and the standard curve was generated. TFC is expressed as mg of quercetin equivalent (mg of QE.g^-1^ of extract).

#### Determination of total polyphenolic content (TPC)

TPC was determined by Folin-Ciocalteu phenol reagent method with slight modifications [64]. Briefly, 0.5 mL of the extract was mixed 2.5 mL of 10% Folin-Ciocalteu’s phenol reagent dissolved in water. After 3 min, 2.5 mL of 7.5% NaHCO_3_ was added. The same procedure was followed for the preparation of the blank sample. The reaction was kept in the dark for 90 min, after which the absorbance was measured at 765 nm. TPC is expressed as mg of gallic acid equivalent (mg of GAE.g ^-1^ of extract).

#### Gas chromatography-mass spectrometry (GC-MS) analysis

A GC-MS analysis was performed by a previously reported method with slight modifications [65]. Briefly, two μL aliquot of freeze-dried and concentrated BRARP extract in chloroform and methanol was analyzed by an Agilent GC-MS 7890A, 5975C (Agilent, USA) to identify the volatile compounds. The column used was HP DB-5 capillary column (30×0.25 mm×0.25 μm; Agilent Technologies). GC oven initial temperature was 50°C for 2 min and programmed to 280°C at a rate of 5°C.min^-1^, and finally held at 280°C for 2 min. Operating conditions for GC as follows: hydrogen was used as carrier gas (5 mL.min^-1^); the temperature of injector and detector was 250°C and 280°C, respectively; the volume injected was 2 μL in split mode (10: 1). The mass spectra were performed at 70 eV of the mass range of 50∼400.

#### Cytotoxicity

**Fractionation** of different solvents including ethyl ether, toluene, ethanol, dichloromethane, chloroform, and methanol was used for fractionation of the species which had shown to be cytotoxic in MTT assay with a similar method as extraction. The fractions were further tested on the same cell lines that had shown to be cytotoxic in the earlier studies.

**The cell line** MCF-7 (ATCC® HTB-22™) (human breast adenocarcinoma, cell type—epithelial) was provided from the School of Food Science and Biotechnology, Kyungpook National University, Daegu, South Korea. Each cell line was cultured in suitable medium to obtain the desired growth and the growth curve of the cell line was plotted.

**Cytotoxicity** of BRARP extracts was performed against MCF-7 cell line by MTT assay according to a method described elsewhere [66–67]. The MCF-7 cell (6×10^3^) was grown in 96- well microtitration plate. Following 24 h incubation, the cells were treated with different concentrations (the maximum concentration was 50 μg.mL^-1^) of the BRARP extracts for 72 h. 3-(4,5-dimethylthiazol-2-yl)-2,5- diphenyl tetrazolium bromide solution (MTT) solution (5 mg.mL^-1^ of final concentration) was added to each well and incubated at 37°C for 4 h. Then the supernatant was removed and the resultant formazan crystals were dissolved in DMSO. The amount of produced formazan is directly proportional to the number of living cells. The absorbance was measured using a microplate reader at 570 nm. MTT assay for the cytotoxicity of commercially available identified compounds was performed similarly as described above for BARP extracts. Tamoxifen was used as the positive control in the present study (% cell viability = A570 of treated cells / A570 of control cells × 100%).

#### Molecular modeling

Computer-based molecular modeling method was adapted to identify possible binding mode and theoretical affinity of molecules derived from BRARP against LpxC. The molecules (*E*)-2-Butenoic acid propyl ester, Phenol, Sodium phenoxide, 4-Pyridinecarboxylic acid, s-Triazolo[4,3-a]pyridine, 1,2-Benzenedicarboxylic acid, 2,2-Dimethoxybutane and 2,3-Dicyanopropionamide were used and ligand structures (metabolites) were prepared by using ACD/ Chem Sketch based on Canonical SMILES obtained from Pub Chem (https://www.ncbi.nlm.nih.gov/pccompound). The crystal structure of protein LpxC [68] was retrieved as PDB file from protein data bank (https://www.wwpdb.org/) and after the energy minimization [69], used as a receptor in molecular modeling. Molecular docking score was calculated using the online molecule system (More than molecule-Docking (Vina); https://mcule.com/) followed by the BIOVIA Discovery Studio 2016 (Accelrys Software Inc., San Diego, CA, USA) to observe the interactions between the protein and ligand. Further, the results of molecular modeling were validated by *in vitro* antimicrobial experiments, in addition to 1, 2-Benzenedicarboxylic acid (Phthalic acid (99% purity) P8657 SIGMA (CAS number 88-99-3).

#### Statistical analysis

All experiments were done in triplicate and replicated at least twice. Results are expressed as the means ± standard error for each group. Statistical analysis was performed by using Student’s t-test. P < 0.05.

## Supporting information

S1 Table Antimicrobial activity of the standard antibiotics against the test microorganisms. (PDF)

S2 Table Effect of the standard chemical preservatives against the microorganisms used in this study. (PDF)

S3 Table Zone of inhibition for pancreatic enzyme treatment. (PDF)

S4 Table Qualitative phytochemical analysis of the leaf and stem extract of BRARP. (PDF)

S5 Table Antimicrobial effect of the compounds present in BRARP extract. (PDF)

S6 Table Cytotoxic activity of standard compounds present in BRARP from South Korea against MCF -7 cell lines. (PDF)

S1 Fig Weaker inhibiting molecules and their molecular interactions. (TIFF)

S2 Fig GC-MS chromatogram of BRARP extract in chloroform. (PDF)

S3 Fig GC-MS chromatogram of BRARP extract in methanol. (PDF)
