# Supplementary material for: Preservative effect of Chinese cabbage (Brassica rapa subsp. pekinensis) extract on their molecular docking, antioxidant and antimicrobial properties
Source: PLoS One. 2018 Oct 3;13(10):e0203306. doi: 10.1371/journal.pone.0203306 (PMC6169867; doi:10.1371/journal.pone.0203306)
Supplement: S1 Table — (PDF) [file pone.0203306.s001.pdf]

S1 Table Antimicrobial activity of the standard antibiotics against the test microorganisms

Classes of antibiotics	Antibiotics (30 μg.mL^{-1})	Zone of inhibition (mm)			
		494 (Isolate)	ATCC 43894	ATCC 35150	ATCC 13150
Cell wall Targeting Antibiotic (A)	Ampicillin	20.00 ± 0.01[a]	20.00 ± 0.03[a]	20.00 ± 0.05[a]	14.00 ± 0.02[b]
	Vancomycin	-	-	-	14.00 ± 0.02[a]
	Penicillin	15.00 ± 0.02[a]	15.00 ± 0.05[a]	15.00 ± 0.01[a]	14.00 ± 0.04[ab]
Protein synthesis inhibiting Targeting Antibiotic (B)	Gentamicin	13.00 ± 0.05[c]	17.00 ± 0.03[a]	15.00 ± 0.04[b]	17.00 ± 0.05[a]
	Tetracycline	16.00± 0.01[d]	19.00 ± 0.03[b]	20.00 ± 0.05[b]	23.00 ± 0.05[a]
	kanamycin	20.00 ± 0.02[a]	20.00 ± 0.05[a]	17.00 ± 0.04[b]	16.00 ± 0.04[c]
	Clindamycin	-	-	12.00 ± 0.03[b]	25.00 ± 0.05[a]
	Erythromycin	-	-	-	13.00 ± 0.06[a]
Nucleic Acid targeting Antibiotic (C)	Novobiocin	10.00 ± 0.05[b]	10.00 ± 0.03[b]	-	25.00± 0.04[a]

-: not active, [a]: more sensitive, [b]: moderate sensitive, [c]: less sensitive, Media – Tryptic soy agar.
