# Supplementary material for: Preservative effect of Chinese cabbage (Brassica rapa subsp. pekinensis) extract on their molecular docking, antioxidant and antimicrobial properties
Source: PLoS One. 2018 Oct 3;13(10):e0203306. doi: 10.1371/journal.pone.0203306 (PMC6169867; doi:10.1371/journal.pone.0203306)
Supplement: S2 Table — (PDF) [file pone.0203306.s002.pdf]

S2 Table Effect of the standard chemical preservatives against the microorganisms used in this study

List of microorganisms	Zone of inhibition (mm)					
	Sodium metabisulfite		Sodium benzoate		Sodium nitrite	
	1 %	5 %	1 %	5 %	1 %	5%
**Gram-negative bacteria**						
494 (Isolate)	09.00 ± 0.03[b]	11.00 ± 0.05[a]	-	-	-	-
ATCC 35150	10.00 ± 0.02[b]	13.00 ± 0.05[a]	-	-	-	-
ATCC 43894	11.00 ± 0.03[b]	13.00± 0.03[a]	-	-	-	-
**Gram-positive bacteria**						
ATCC 13150	11.00 ± 0.01[a]	11.00 ± 0.04[a]	-	-	-	-
KCTC 21004	10.00 ± 0.04[a]	10.00 ± 0.03[a]	-	-	-	-
KCTC 3545	10.00 ± 0.02[a]	11.00 ± 0.03[a]	-	-	-	-
KCTC 13302	10.00 ± 0.05[a]	10.00± 0.03[a]	-	-	-	-
**Fungi**						
KCTC 7965	-	-	-	-	-	-
KCTC 6145	10.00 ± 0.03[a]	10.00 ± 0.03[a]	-	-	-	-
KCTC 6143	10.00 ± 0.05[a]	10.00 ± 0.04[a]	-	-	-	-
KCTC 6317	10.00 ± 0.01[a]	10.00 ± 0.03[a]	-	-	-	-

-: not active, [a]: more sensitive, [b]: moderate sensitive, [c]: less sensitive, Media – Tryptic soy agar.
