# Supplementary material for: Preservative effect of Chinese cabbage (Brassica rapa subsp. pekinensis) extract on their molecular docking, antioxidant and antimicrobial properties
Source: PLoS One. 2018 Oct 3;13(10):e0203306. doi: 10.1371/journal.pone.0203306 (PMC6169867; doi:10.1371/journal.pone.0203306)
Supplement: S3 Table — (PDF) [file pone.0203306.s003.pdf]

S3 Table Zone of inhibition for pancreatic enzyme treatment

**List of microorganisms**	**Zone of inhibition (mm)**						
**BRARP extracts (Conc. 33mg.mL^{-1})**	**CE**	**TE**	**DE**	**EEE**	**EtE**	**ME**	**DWE**
**Gram-negative bacteria**							
494 (Isolate)	13.00 ± 0.01^{a}	12.00 ± 0.03^{bc}	12.30 ± 0.05^{b}	12.30 ± 0.05^{b}	-	-	-
ATCC 35150	13.00 ± 0.03^{a}	12.00 ± 0.05^{b}	12.00 ± 0.01^{b}	13.00 ± 0.04^{a}	-	-	-
ATCC 43894	13.00 ± 0.02^{a}	-	12.00 ± 0.05^{b}	11.00 ± 0.03^{c}	-	-	-
**Gram-positive bacteria**							
ATCC 13150	10.00 ± 0.01^{b}	12.00 ± 0.05^{a}	10.00± 0.03^{b}	10.00 ± 0.04^{b}	-	-	-
KCTC 21004	10.00± 0.03^{a}	-	10.00± 0.05^{a}	09.30 ± 0.03^{b}	-	-	-
KCTC 3545	11.00± 0.02^{a}	-	11.00 ± 0.03^{a}	10.00 ± 0.05^{b}	-	-	-
KCTC 13302	10.00 ± 0.02^{a}	-	09.00 ± 0.04^{b}	10.00 ± 0.05^{a}	-	-	-
**Fungi**							
KCTC 7965	10.30 ± 0.03^{a}	-	10.00± 0.05^{ab}	10.30± 0.05^{a}	-	-	-
KCTC 6145	08.80± 0.01^{a}	-	08.30 ± 0.03^{ab}	08.50± 0.03^{ab}	-	-	-
KCTC 6143	-	-	-	-	-	-	-
KCTC 6317	12.50± 0.03^{a}	-	11.50± 0.05^{b}	11.60± 0.03^{b}	-	-	-

-: not active, CE: Chloroform Extract, TE: Toluene Extract, DE: Dichloromethane Extract, EEE: Ethyl Ether Extract, EtE: Ethanol Extract, ME: Methanol Extract, DWE: Distilled Water Extract

^{a}: more sensitive, ^{b}: moderate sensitive, ^{c}: less sensitive, Media – Tryptic soy Broth.
