# Supplementary material for: Preservative effect of Chinese cabbage (Brassica rapa subsp. pekinensis) extract on their molecular docking, antioxidant and antimicrobial properties
Source: PLoS One. 2018 Oct 3;13(10):e0203306. doi: 10.1371/journal.pone.0203306 (PMC6169867; doi:10.1371/journal.pone.0203306)
Supplement: S4 Table — (PDF) [file pone.0203306.s004.pdf]

S4 Table Qualitative phytochemical analysis of the leaf and stem extract of BRARP

Sr. No	Phytocompound	CE	DE	EEE	TE	EtE	ME	DWE
1	Phenol	-	-	-	-	-	-	-
2	Saponin	+	+	-	-	-	-	-
3	Glycosides	+	+	+	+	-	-	-
4	Steroids	-	-	+	+	-	-	-
5	Terpenoids	-	-	-	-	-	-	-
6	Tanins	-	-	-	-	-	-	-
7	Flavonoid	+	+	+	+	-	-	-

-: not present, +: present, CE: Chloroform Extract, TE: Toluene Extract, DE: Dichloromethane Extract, EEE: Ethyl Ether Extract, EtE: Ethanol Extract, ME: Methanol Extract, DWE: Distilled Water Extract
