# Supplementary material for: Preservative effect of Chinese cabbage (Brassica rapa subsp. pekinensis) extract on their molecular docking, antioxidant and antimicrobial properties
Source: PLoS One. 2018 Oct 3;13(10):e0203306. doi: 10.1371/journal.pone.0203306 (PMC6169867; doi:10.1371/journal.pone.0203306)
Supplement: S5 Table — (PDF) [file pone.0203306.s005.pdf]

S5 Table Antimicrobial effect of the compounds present in BRARP extract

List of microorganisms	Zone of inhibition (mm)					
	(E)-2-Butenoic acid propyl ester		Phenol		Sodium phenoxide	
	0.5 $mg.mL^{-1}$	1.0 $mg.mL^{-1}$	0.5 $mg.mL^{-1}$	1.0 $mg.mL^{-1}$	0.5 $mg.mL^{-1}$	1.0 $mg.mL^{-1}$
**Gram-negative bacteria**						
494 (Isolate)	-	-	-	-	-	-
ATCC 35150	-	-	-	-	-	-
ATCC 43894	-	-	-	-	-	-
**Gram-positive bacteria**						
ATCC 13150	-	-	-	-	-	-
KCTC 21004	-	-	-	-	-	-
KCTC 3545	-	-	-	-	-	-
KCTC 13302	-	-	-	-	-	-
**Fungi**						
KCTC 7965	-	-	-	-	-	-
KCTC 6145	-	-	-	-	-	-
KCTC 6143	-	-	-	-	-	-
KCTC 6317	-	-	-	-	-	-

List of microorganisms	Zone of inhibition (mm)					
	4-Pyridinecarboxylic acid		1,2-Benzenedicarboxylic acid (Phthalic acid)		s-Triazolo[4, 3-a]pyridazine	
	0.5 $mg.mL^{-1}$	1.0 $mg.mL^{-1}$	0.5 $mg.mL^{-1}$	1.0 $mg.mL^{-1}$	0.5 $mg.mL^{-1}$	1.0 $mg.mL^{-1}$
**Gram-negative bacteria**						
494 (Isolate)	-	-	09.00 ± 0.03[b]	15.00 ± 0.01[a]	09.00 ± 0.03[b]	11.00 ± 0.05[a]
ATCC 35150	-	-	10.00 ± 0.02[b]	15.00 ± 0.04[b]	-	-
ATCC 43894	-	-	11.00 ± 0.03[b]	20.00 ± 0.05[b]	-	-
**Gram-positive bacteria**						
ATCC 13150	-	-	11.00 ± 0.01[a]	15.00 ± 0.05[a]	-	-
KCTC 21004	-	-	10.00 ± 0.04[a]	17.00 ± 0.03[a]	11.00 ± 0.01[a]	11.00 ± 0.04[a]
KCTC 3545	-	-	10.00 ± 0.02[a]	19.00 ± 0.03[b]	10.00 ± 0.04[a]	10.00 ± 0.03[a]
KCTC 13302	-	-	10.00 ± 0.05[a]	20.00 ± 0.05[a]		
**Fungi**						
KCTC 7965	-	-	11.00 ± 0.01[a]	11.00 ± 0.05[a]	-	-
KCTC 6145	-	-	10.00 ± 0.04[a]	13.00 ± 0.05[a]	-	-
KCTC 6143	-	-	10.00 ± 0.04[a]	13.00± 0.03[a]	-	-
KCTC 6317	-	-	10.00 ± 0.03[a]	11.00 ± 0.05[a]	-	-

List of microorganisms	Zone of inhibition (mm)					
	2,2-Dimethoxybutane		2,3-Dicyanopropionamide		DMSO	
	0.5 mg.mL^{-1}	1.0 mg.mL^{-1}	0.5 mg.mL^{-1}	1.0 mg.mL^{-1}	0.5 mg.mL^{-1}	1.0 mg.mL^{-1}
**Gram-negative bacteria**						
494 (Isolate)	09.00 ± 0.03[b]	11.00 ± 0.05[a]	-	-	-	-
ATCC 35150	10.00 ± 0.02[b]	13.00 ± 0.05[a]	-	-	-	-
ATCC 43894			-	-	-	-
**Gram-positive bacteria**						
ATCC 13150	-	-	-	-	-	-
KCTC 21004	-	-	-	-	-	-
KCTC 3545	-	-	-	-	-	-
KCTC 13302	10.00 ± 0.05[a]	10.00± 0.03[a]	-	-	-	-
**Fungi**						
KCTC 7965	-	-	-	-	-	-
KCTC 6145	-	-	-	-	-	-
KCTC 6143	-	-	-	-	-	-
KCTC 6317	-	-	-	-	-	-

-: not active, [a]: more sensitive, [b]: moderate sensitive, [c]: less sensitive, Media – Tryptic soy agar.
