# Supplementary material for: Preservative effect of Chinese cabbage (Brassica rapa subsp. pekinensis) extract on their molecular docking, antioxidant and antimicrobial properties
Source: PLoS One. 2018 Oct 3;13(10):e0203306. doi: 10.1371/journal.pone.0203306 (PMC6169867; doi:10.1371/journal.pone.0203306)
Supplement: S6 Table — (PDF) [file pone.0203306.s006.pdf]

S6 Table. Cytotoxic activity of standard compounds present in BRARP from South Korea against MCF -7 cell lines

Sr. No	Standard chemical Compound Identified in BRARP	IC_{50} (μg.mL^{-1}) MCF-7	IC_{50} (μg.mL^{-1}) MCF-7
		**24h**	**48h**
1	(E)-2-Butenoic acid propyl ester	>50	>50
2	Phenol	>50	>50
3	Sodium phenoxide	>50	>50
4	4-Pyridinecarboxylic acid	>50	>50
5	s-Triazolo[4,3-a]pyridine	>50	>50
6	1,2-Benzenedicarboxylic acid (Phthalic acid)	>50	44.3 ± 1.1
7	2,2-Dimethoxybutane	>50	>50
8	2,3-Dicyanopropionamide	>50	41 ± 1.7

-: IC_{50} (inhibitory concentration 50 %), MCF: Michigan Cancer Foundation-7 (Brest cancer cell line)

Name the compound	Chemical formula	Catalog Number	Company	Cytotoxic effect
**Chloroform extract**				
(E)-2-Butenoic acid propyl ester	$C_7H_{12}O_2$	751162 CAS# 2210-28-8	Sigma-Aldrich	NCT
Phenol	C_6H_6O	P1037 CAS# 108-95-2	Sigma-Aldrich	NCT
Sodium phenoxide	C_6H_5NaO	CDS001581 CAS# 139-02-6	Sigma-Aldrich	NCT
4-Pyridinecarboxylic acid	$C_6H_5NO_2$	8.00736 CAS# 55-22-1	Sigma-Aldrich	NCT
s-Triazolo[4,3-a]pyridine	$C_6H_5N_3$	CAS# 069872	Matrix Scientific	NCT
1,2-Benzenedicarboxylic acid (Phthalic acid)	$C_8H_6O_4$	79255 CAS# 88-99-3	Sigma-Aldrich	MCT
**Methanol extract**				
2,2-Dimethoxybutane	$C_6H_{14}O_2$	Catalog#125785 CAS# 3453-99-4	Matrix science	NT
2,3-Dicyanopropionamide	$C_5H_5N_3O$	40751 CAS# 98-96-4	Sigma-Aldrich	MCT

-: NCT - Non cytotoxic; MCT – Moderate cytotoxic effect
