# Supplementary material for: Preservative effect of Chinese cabbage (Brassica rapa subsp. pekinensis) extract on their molecular docking, antioxidant and antimicrobial properties
Source: PLoS One. 2018 Oct 3;13(10):e0203306. doi: 10.1371/journal.pone.0203306 (PMC6169867; doi:10.1371/journal.pone.0203306)
Supplement: S2 Fig — (PDF) [file pone.0203306.s008.pdf]

Chinese cabbage chloroform extract

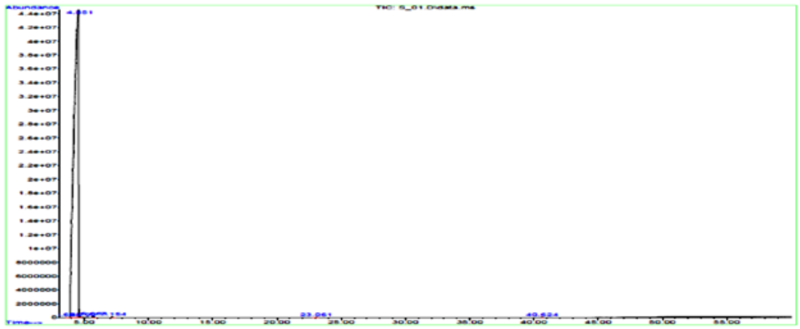

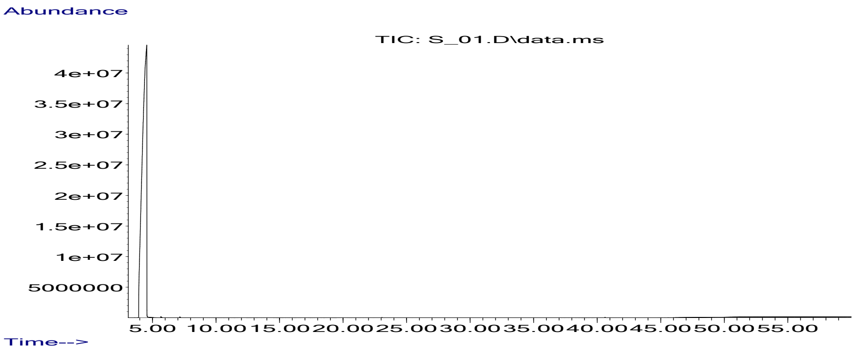

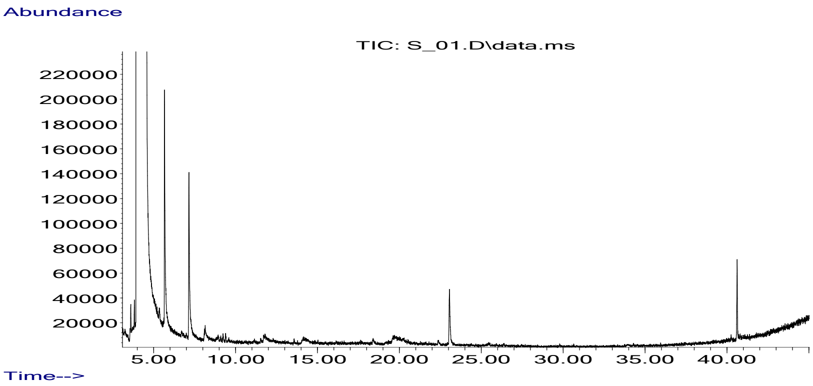

Peak	R.T time	Type	Width	Area	Start time	End time
1	3.605	VV	0.027	425422	3.578	3.654
2	4.551	PV	0.288	1.079E+10	3.86	4.781
3	4.792	VV	0.023	117303	4.781	4.816
4	5.663	VV	0.045	6097082	5.637	5.937
5	7.154	PV	0.05	5035212	7.105	7.387
6	23.061	BV	0.054	1823455	22.845	23.221
7	40.624	BV	0.034	1588487	40.535	40.688

Library report - C:\Database\W8N05ST.L

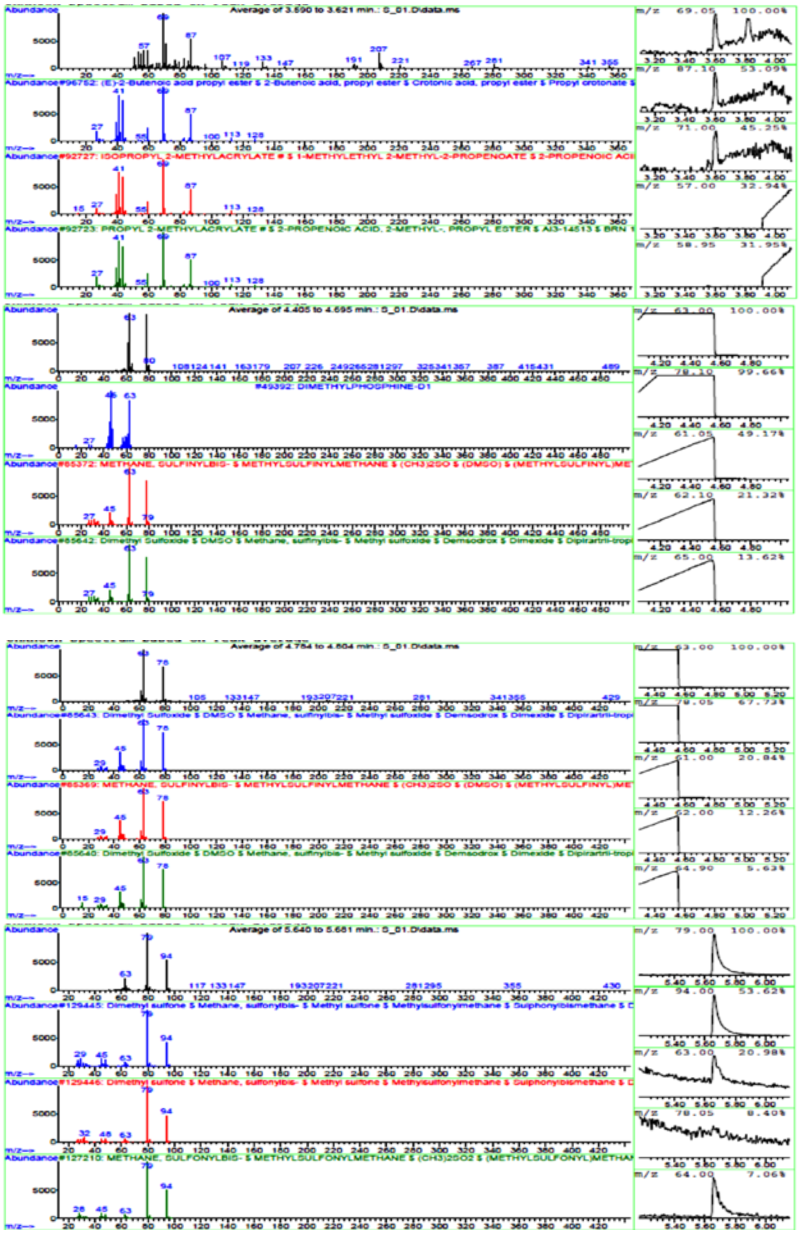

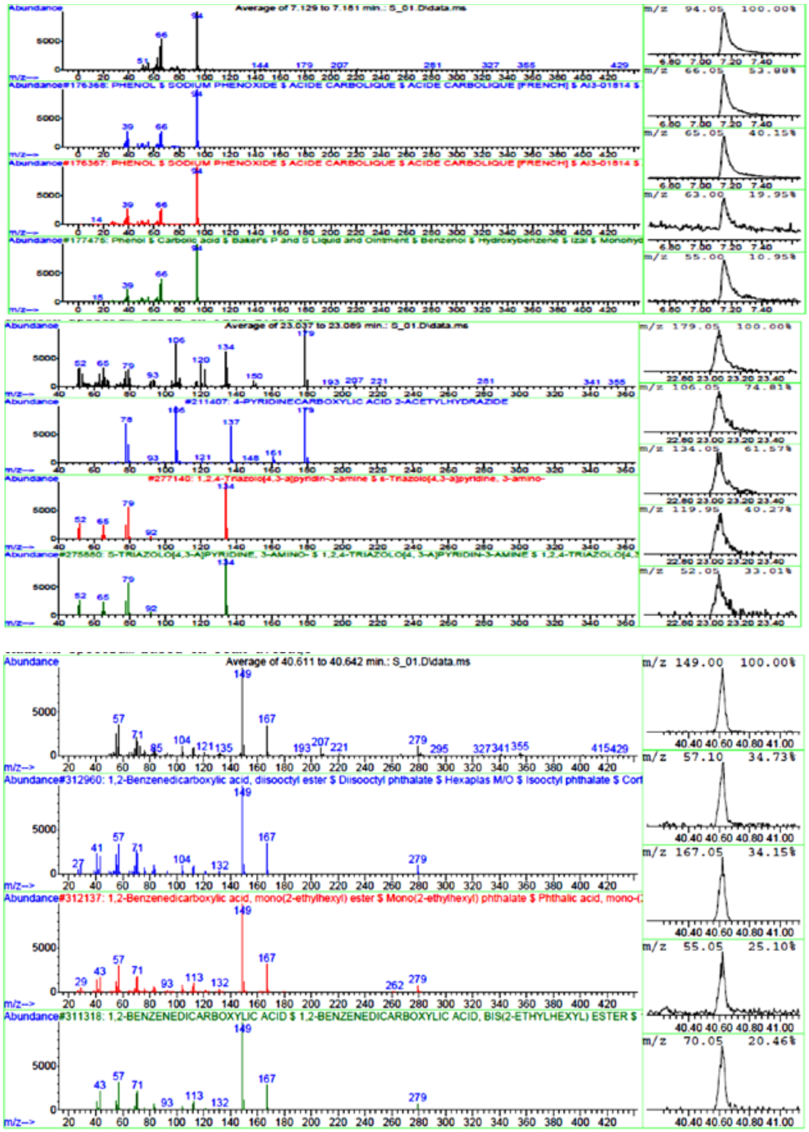

S2 Fig. GC-MS chromatogram of BRARP extract in chloroform
