# Supplementary material for: Preservative effect of Chinese cabbage (Brassica rapa subsp. pekinensis) extract on their molecular docking, antioxidant and antimicrobial properties
Source: PLoS One. 2018 Oct 3;13(10):e0203306. doi: 10.1371/journal.pone.0203306 (PMC6169867; doi:10.1371/journal.pone.0203306)
Supplement: S3 Fig — (PDF) [file pone.0203306.s009.pdf]

Chinese cabbage methanol extract

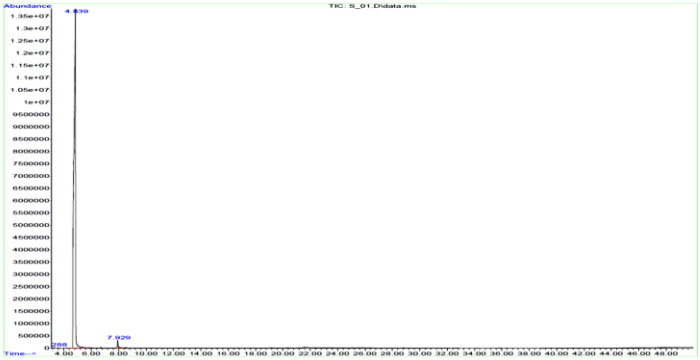

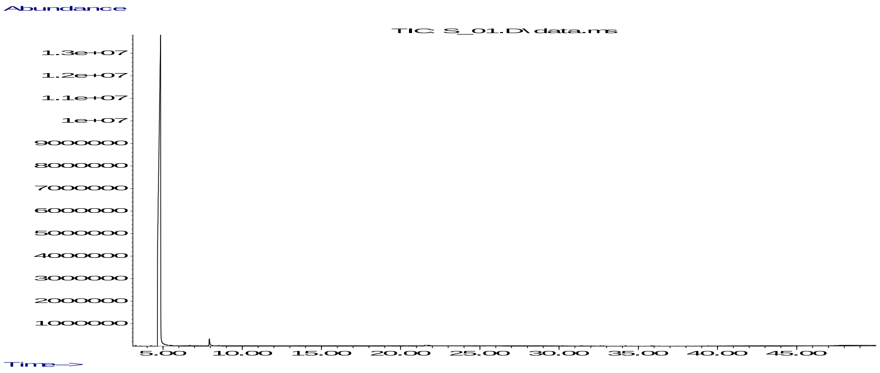

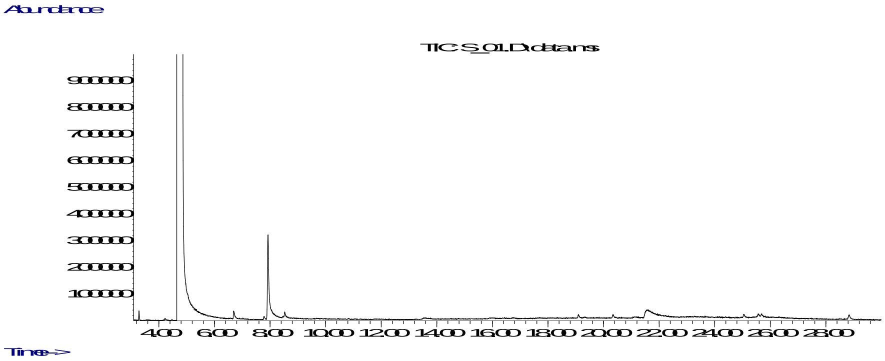

Peak	R.T time	Type	Width	Area	Start time	End time
1	3.288	BB	0.043	530502	3.247	3.347
2	4.839	BB	0.111	1.166E+09	4.422	5.604
3	7.929	PB	0.064	14000713	7.862	8.337

Library report - C:\Database\W8N05ST.L

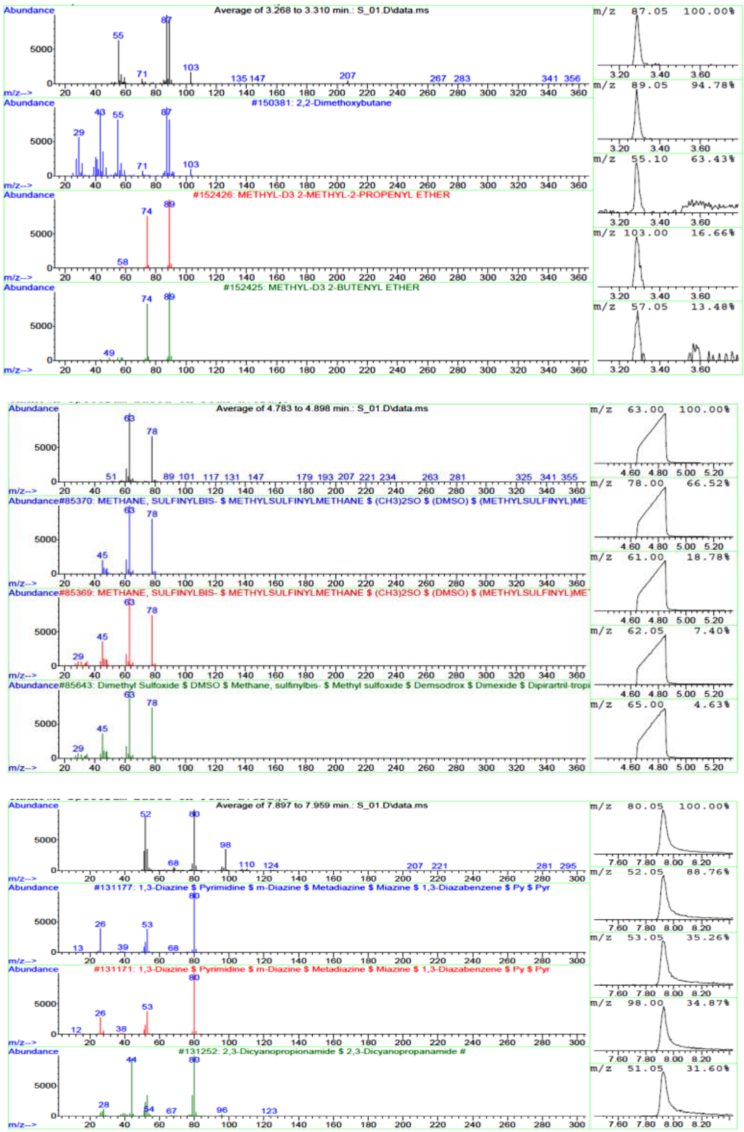

S3 Fig. GC-MS chromatogram of BRARP extract in methanol
